# Constrained parsimonious model-based clustering

**DOI:** 10.1007/s11222-021-10061-3

**Published:** 2021-11-20

**Authors:** Luis A. García-Escudero, Agustín Mayo-Iscar, Marco Riani

**Affiliations:** 1 Department of Statistics and Operational Research and IMUVA, University of Valladolid, Valladolid, Spain; 2 Department of Economics and Management and Interdepartmental Centre of Robust Statistics, University of Parma, Parma, Italy

**Keywords:** Model-based clustering, Mixture modeling, Constraints

## Abstract

A new methodology for constrained parsimonious model-based clustering is introduced, where some tuning parameter allows to control the strength of these constraints. The methodology includes the 14 parsimonious models that are often applied in model-based clustering when assuming normal components as limit cases. This is done in a natural way by filling the gap among models and providing a smooth transition among them. The methodology provides mathematically well-defined problems and is also useful to prevent us from obtaining spurious solutions. Novel information criteria are proposed to help the user in choosing parameters. The interest of the proposed methodology is illustrated through simulation studies and a real-data application on COVID data.

## Introduction

Model-based clustering is a well-established and powerful approach to cluster analysis. Fitting *k* multivariate Gaussian distributed components to data is the most widely applied methodology and maximum likelihood is the principle often adopted for the fitting procedure.

In this work, we use the notation $\phi (\cdot ; \mu , \varSigma )$ for the probability density functions of the *p*-variate normal distribution with mean $\mu$ and covariance matrix $\varSigma$. Given a sample of *p*-dimensional observations $\{ x_1,\cdots , x_n\}$, the *classification likelihood* approach searches for a partition $\{H_1,\ldots ,H_k\}$ of the $\{1,\cdots ,n\}$ indices, mean vectors $\mu _1,\cdots , \mu _k$ in ${\mathbb {R}}^p$, symmetric positive semidefinite $p\times p$ scatter matrices $\varSigma _1,\cdots , \varSigma _k$ and positive weights $\pi _1,\cdots ,\pi _k$ with $\sum _{j=1}^k \pi _j=1$, which maximizes

1 $$\begin{aligned} \sum _{j=1}^k\sum _{i\in H_j} \log \left( \pi _j\phi ( x_i; \mu _j, \varSigma _j)\right) . \end{aligned}$$

Alternatively, the *mixture likelihood* approach seeks the maximization of

2 $$\begin{aligned} \sum _{i=1}^n \log \left( \sum _{j=1}^k\pi _j\phi ( x_i; \mu _j, \varSigma _j)\right) . \end{aligned}$$

An important problem when maximizing (1) and (2) is that these two target likelihood functions are unbounded ones (Kiefer and Wolfowitz 1956; Day 1969). Another important issue is the typically large number of local maxima that can be found. In the mixture likelihood case, the existence of a sequence of local maxima converging to the true mixture parameters is guaranteed as the sample size *n* increases. However, it is not obvious how to choose those local maxima in practical applications. In fact, many local maxima related to these very high values of the likelihoods are known to be clearly non-interesting and often referred to as “spurious solutions” (see, e.g., chapter 3.10 in McLachlan and Peel (2000)). In these cases, components basically defined from a few, almost collinear, observations are obtained. Algorithms applied for maximizing the target likelihood (EM algorithms when maximizing (1) and CEM algorithm when maximizing (2)) can be affected by unboundedness, being trapped into sub-optimal maxima or detect non-interesting local maxima. This is even more problematic when applying well-known information criteria (such as BIC and ICL). These criteria are based on penalized versions of the target likelihood values and spurious solutions or the unboundedness issue can result in artificially large values for the likelihood. Note also that it is necessary, when choosing *k*, to fit models with a higher than needed number of components. All the previously mentioned problems are even more likely there to appear.

These problems with local maxima can be in principle solved by carefully exploring and analyzing *all* possible local maxima (McLachlan and Peel 2000). Although some interesting procedures have been introduced in that direction (see, e.g., Gallegos and Ritter (2018)), this approach is not straightforward and is certainly time consuming. Another widely applied remedy consists in trying to initialize the algorithms adequately in order that iterations return good local maxima. It is well-known that EM and CEM algorithms are highly dependent on their initialization, but it is also true that adequate initialization strategies (for instance, appropriate hierarchical model-based clustering initializations) often result in sensible local maxima. However, theoretical guarantees about correctness of initializations are difficult to establish and it may happen that the final fitted model inherits significant drawbacks from the initializing procedure. Additionally, if two different initializations provide different final results, it is difficult to justify not choosing the one with the larger value of the likelihood without any further analysis. In fact, some initialization procedures are clearly aimed at searching directly for the largest values (see, e.g. Biernacki et al. 2003).

It is also common to enforce constraints on the $\varSigma _j$ scatter matrices when maximizing (1) or (2). Among them, the use of “parsimonious” models (Celeux and Govaert 1995; Banfield and Raftery 1993) is one of the most popular and widely applied approaches in practice. These parsimonious models follow from a decomposition of the $\varSigma _j$ scatter matrices as

3 $$\begin{aligned} \varSigma _j=\lambda _j \varOmega _j \varGamma _j \varOmega _j', \end{aligned}$$

with $\lambda _j=|\varSigma _j|^{1/p}$ (volume parameters),

$$\begin{aligned} \varGamma _j&=\mathsf {diag}(\gamma _{j1},\ldots ,\gamma _{jl},\ldots ,\gamma _{jp})\text { with }\\ \mathsf {det}(\varGamma _j)&=\prod _{l=1}^p \gamma _{jl}=1 \end{aligned}$$

(shape matrices), and $\varOmega _j$ (rotation matrices) with $\varOmega _j\varOmega _j'=\textsf {I}_p$. Different constraints on the $\lambda _j$, $\varOmega _j$ and $\varGamma _j$ elements are considered across components to get 14 parsimonious models (which are coded with a combination of three letters). These models reduce notably the number of free parameters to be estimated, so improving efficiency and model interpretability. Moreover, many of them turn the constrained maximization of the likelihoods into well-defined problems and help to avoid spurious solutions. Unfortunately, the problems remain for models with unconstrained $\lambda _j$ volume parameters, which are coded with the first letter as a V (V** models). Aside from relying on good initializations, it is common to consider the early stopping of iterations when approaching scatter matrices with very small eigenvalues or when detecting components accounting for a reduced number of observations. A not fully iterated solution (or no solution at all) is then returned in these cases. The idea is known to be problematic when dealing with (well-separated) components made up of a few observations.

Starting from a seminal paper by Hathaway (1985), an alternative approach is to constrain the $\varSigma _j$ scatter matrices by specifying some tuning constants that control the strength of the constraints. A fairly comprehensive review of this approach can be found in García-Escudero et al. (2018). For instance, a recent proposal following this idea is the “deter-and-shape” one in García-Escudero et al. (2020). The maximal ratio among the $\lambda _j$ terms is there constrained to be smaller than a fixed constant and, additionally, the maximal ratio $\gamma _{jl}/\gamma _{jl'}$ in each $\varGamma _j$ shape matrix is also constrained to be smaller than another fixed constant. In this work, we will refer to these second type of constraint as “shape-within” as they control the relative sizes of the shape elements “within” each shape matrix individually. When this second constant is set equal to 1, since all $\gamma _{jl}$ are then equal 1, we are imposing spherical components.

**Fig. 1:**
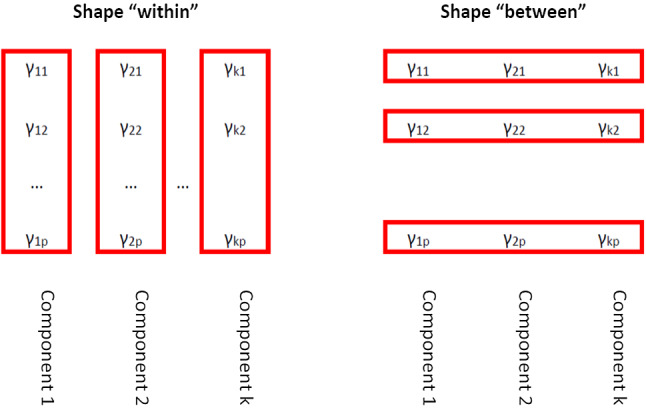
Summary of the types of constraints considered on the shape elements

In this work, we introduce new “shape-between” constraints where the maximal ratio $\gamma _{jl}/\gamma _{j'l}$ is controlled for every fixed *l* for $l=1,\ldots ,p$. Figure 1 shows a summary of the two types of constraints considered on the shape elements. Notice that we have $\gamma _{jl}=\gamma _{j'l}$ for every *j* and $j'$ in the most constrained case, but the fitted components are not necessarily spherical. Therefore, these new constraints are better suited to control differences among shape matrices without assuming sphericity. The new constraints can be easily combined with others typically imposed on the $\varOmega _j$ rotation matrices.

The main contributions of this work are the following:

- The proposed constraints yield well-defined problems and it is not necessary to include the specification of any particular initialization strategy. An underlying population (theoretical) problem can thus be defined. Section 4 shows existence results for both the sample and the population problems and we prove the consistency for the sample solutions to the population one.
- The new constraints allow us to achieve, as limit cases, the 14 parsimonious models which are commonly applied in model-based clustering. These popular parsimonious models cannot be obtained as limit cases when only considering “deter-and-shape(within)” constraints or other constraints such as the ones based on eigenvalues ratios (García-Escudero et al. 2015). However, contrary to what happens with V** models, the associated likelihood maximization problems are always well defined. It is perhaps too extreme to choose only among strongly constrained models (maximal ratios exactly equal to 1) and the fully unconstrained ones (maximal ratios taking arbitrarily large values). Sometimes it is clear that data do not suggest considering the most constrained models, but leaving them fully unrestricted may cause estimation instabilities and the detection of spurious solutions. A smooth transition between these extreme cases can be obtained with the proposed methodology. An interesting connection between the two types of constraints (between-within) in the shape matrices elements is given in Sect. 2, together with some practical consequences.
- Although 14 different algorithms are often employed to estimate the classical parsimonious models, a unifying algorithm is proposed in Sect. 3 which includes all the 14 classical parsimonious models as limit cases.
- Some general guidelines about how to choose the tuning parameters are provided. In fact, the smooth transition among models turns out to be useful to introduce novel information criteria, inspired by those in Cerioli et al. (2018). These criteria penalize high likelihood values resulting from unnecessary model complexity associated with the constraints. Model complexity here does not necessarily correspond to the total number of parameters, but it simply means that more flexibility in the constraints allows us to fit more varied models. This proposal can be seen as a first step in order to obtain a reduced list of “sensible” cluster solutions, as done in Cerioli et al. (2018).

Some simulations and a real data example are provided in Sects. 6 and 7 to illustrate the interest of the proposed methodology. We do not claim that the well-established and widely applied proposals considered for comparison are useless; they have amply demonstrated their usefulness. However, we illustrate that the proposed methodology can also be very useful and that there is room for further investigations of this new proposal. Concluding remarks and open research directions are given in Sect. 8.

## Proposed methodology

We impose three different types of constraints on the $\varSigma _j$ matrices which depend on three constants $c_{\mathsf {det}}$, $c_{\mathsf {shw}}$ and $c_{\mathsf {shb}}$ all of them being greater than or equal to 1.

The first type of constraint serves to control the maximal ratio among determinants and, consequently, the maximum allowed difference between component volumes:

4 $$\begin{aligned} \textsf {``deter'': }\qquad \frac{\max _{j=1,\ldots ,k} |\varSigma _j|}{\min _{j=1,\ldots ,k} |\varSigma _j|}=\frac{\max _{j=1,\ldots ,k} \lambda _j^{p}}{\min _{j=1,\ldots ,k} \lambda _j^{p}}\le c_{\mathsf {det}}.\nonumber \\ \end{aligned}$$

The second type of constraint controls departures from sphericity “within” each component:

5 $$\begin{aligned} \textsf {shape-``within'': }&\,\,\, \frac{\max _{l=1,\ldots ,p} \gamma _{jl}}{\min _{l=1,\ldots ,p} \gamma _{jl}}\nonumber \\&\le c_{\mathsf {shw}}\text { for }j=1,\ldots ,k. \end{aligned}$$

This provides a set of *k* constraints that in the most constrained case, $c_{\mathsf {shw}}=1$, imposes $\varGamma _1= \cdots =\varGamma _p=\textsf {I}_p$, where $\textsf {I}_p$ is the identity matrix of size *p*, i.e., sphericity of components.

Constraints (4) and (5) were the basis for the “deter-and-shape” constraints in García-Escudero et al. (2020). These two constraints resulted in mathematically well-defined constrained maximizations of the likelihoods in (1) and (2). However, although highly operative in many cases, they do not include, as limit cases, all the already mentioned 14 parsimonious models. For instance, we may be interested in the same (or not very different) $\varGamma _j$ or $\varSigma _j$ matrices for all the mixture components and these cannot be obtained as limit cases from the “deter-and-shape” constraints.

In this work, we introduce a third type of constraint that serves to control the maximum allowed difference between shape elements “between” components:

6 $$\begin{aligned} \textsf {shape-``between'': }&\,\,\, \frac{\max _{j=1,\ldots ,k} \gamma _{jl}}{\min _{j=1,\ldots ,k} \gamma _{jl}}\nonumber \\&\le c_{\mathsf {shb}}\text { for }l=1,\ldots ,p. \end{aligned}$$

This new type of constraint allows us to impose “similar” shape matrices for the components and, consequently, enforce $\varGamma _1=\cdots =\varGamma _k$ in the most constrained $c_{\mathsf {shb}}=1$ case .

Additionally, another type of constraint on the rotation $\varOmega _j$ matrices can be combined with the previous ones. Three different constraints rot on the rotation matrices can be considered and coded with the letters E, I and V. If rot=E, then we are assuming the same rotation matrices $\varOmega _1=\cdots =\varOmega _k$ for all the components. If rot=I, then we are assuming $\varOmega _1=\cdots =\varOmega _k={\mathsf {I}}_p$, i.e. axes parallel to the coordinate axes (conditional independence within cluster components). Finally, rot=V leaves the rotation matrices $\varOmega _j$ fully unconstrained.

In the third case of fully unconstrained rotation matrices rot=V, we choose the diagonal elements of $\varGamma _j$ (by choosing the appropriate rotation $\varOmega _j$ matrices) such that these shape elements appear in non-increasing order:

7 $$\begin{aligned} \gamma _{j1}\ge \cdots \ge \gamma _{jl}\ge \cdots \ge \gamma _{jp}. \end{aligned}$$

This ordering makes sense since adequate rotations (in the rot=V case) can be performed such that ordered elements within each shape matrix are achieved.

The following lemma shows an interesting connection between the two different types of constraints on the shape matrices.

### Lemma 1

If the “shape-within” constraints (5) are satisfied for a constant $c_{\mathsf {shw}}\ge 1$, then

8 $$\begin{aligned} \frac{\gamma _{jl}}{\gamma _{j'l}}\le c_{\mathsf {shw}}^{(p-1)/p}, \end{aligned}$$

for any $j,j'\in \{1,\ldots ,k\}$ and $l=1,\ldots ,p$.

The proof of this technical lemma is given in Appendix A. When taking into account the definition of the “shape-between” constraints in (6) as a maximal ratio, the previous lemma implies that the choice of $c_{\mathsf {shw}}$ in (5) modifies the effect of $c_{\mathsf {shb}}$ in (6) and that there is no point in considering $c_{\mathsf {shb}}$ not obeying

9 $$\begin{aligned} c_{\mathsf {shb}}\le c_{\mathsf {shw}}^{(p-1)/p}. \end{aligned}$$

For instance, this implies that $c_{\mathsf {shb}}\le \sqrt{c_{\mathsf {shw}}}$ when we are in dimension $p=2$ and that we are obviously assuming $c_{\mathsf {shb}}=1$ whenever we set $c_{\mathsf {shw}}=1$.

An important consequence is that, although we potentially have $2^3\times 3=24$ different extreme models (appearing when $c_{\mathsf {det}}$, $c_{\mathsf {shw}}$ and $c_{\mathsf {shb}}$ are chosen equal to 1 or $\infty$ and the three possible constraints rot on the rotations), not all these 24 models are feasible (because $c_{\mathsf {shw}}=1$ necessarily implies $c_{\mathsf {shb}}=1$) and only the 14 well-known parsimonious models make sense. Table 1 shows how these 14 limit models are derived from different combinations of constraints (this table only includes 14 rows). Table 1 helps to understand the smooth transition among all these 14 models when constants $c_{\mathsf {det}}$, $c_{\mathsf {shw}}$ and $c_{\mathsf {shb}}$ are moved in a controlled fashion. This smooth transition is useful for introducing the novel information criteria in Sect. 5.

**Table 1:** Extreme models for the different limiting values of constants $c_{\mathsf {det}}$, $c_{\mathsf {shw}}$ and $c_{\mathsf {shb}}$ and the three possible rotations in rot

$c_{\mathsf {det}}$	$c_{\mathsf {shw}}$	$c_{\mathsf {shb}}$	rot	Model
1	1	1		EII
	$\infty$	1	I	EEI
			E	EEE
			V	EEV
		$\infty$	I	EVI
			E	EVE
			V	EVV
$\infty$	1	1		VII
	$\infty$	1	I	VEI
			E	VEE
			V	VEV
		$\infty$	I	VVI
			E	VVE
			V	VVV

The “deter-and-shape” constraints also appear as a limit cases when $c_{\mathsf {shb}}$ tends to $\infty$ and rot=V is chosen. Notice that Lemma 1 implies that when $c_{\mathsf {shw}}$ is chosen close to 1 in the “deter-and-shape” approach, then we are also (implicitly) assuming that $c_{\mathsf {shb}}$ is close to 1 too. On the contrary, a large $c_{\mathsf {shw}}$ is still compatible with a $c_{\mathsf {shb}}$ as close to 1 as desired. In fact, choosing moderate values for $c_{\mathsf {det}}$ and $c_{\mathsf {shb}}$ (but not exactly equal to 1) and fixing a very large $c_{\mathsf {shw}}$ value, together with rot=V, turns out to be convenient and advisable, providing a very competitive procedure in many cases.

## Algorithm

In this section, we introduce a feasible ECM algorithm (Meng and Rubin 1993) that can be applied to the proposed methodology. This algorithm covers all the 14 classical parsimonious models as limit cases in a unified fashion and therefore we do not need to consider 14 different algorithms. The “optimal truncation” operator introduced in Fritz et al. (2013), denoted as $\textsf {opt.trunc}$, plays a very important role, as it has in previous constrained model-based clustering approaches. For the sake of completeness, this operator is reviewed in Appendix B.

The proposed algorithm follows analogous steps as EM and CEM algorithms, but iterative procedures may be needed at some points. In the *t*-th step, the constrained scatter matrices are going to be updated as

$$\begin{aligned} \varSigma _j^{(t)}=d_j R_j D_j R_j', \end{aligned}$$

for some $d_j>0$, diagonal matrices $D_j$ with $|D_j|=1$ and $R_j$ being orthogonal matrices. All these $d_j$, $R_j$ and $D_j$ elements are determined through iterative procedures where, roughly speaking, these elements are sequentially improved in turn by optimally updating one of them (in the sense of increasing the target likelihood and fulfilling the constraints) conditionally on the other elements. Further iterations are sometimes required for $D_j$ and $R_j$ within that iterative procedure.

Iterations can be stopped when reaching a maximal number of iterations and we use the notation “iter.max.***” when referring to maximal number of allowed iterations. Additionally, it is useful to monitor “relative changes” in updated parameters and stop iterations whenever relative changes are found smaller than some pre-specified small tolerances. We use the simplified notation $\varDelta b$ for measuring the relative change in the *h*-th iteration of parameter *b* denoted by $b^{(h)}$ with respect to $b^{(h-1)}$ in the previous $(h-1)$-th step (we simplify the notation by deleting the dependence on index *h*). All these small tolerances are going to be notated as “tol.***.” More details on these aspects are provided in Remark 1. Additionally, nstart controls the total number of random initializations.

We denote the parameters at the *t*-th step of the proposed algorithm by $\theta ^{(t)}=(\pi _1^{(t)},\cdots ,\pi _k^{(t)}, \mu _1^{(t)},\cdots , \mu _k^{(t)}, \varSigma _1^{(t)},\cdots , \varSigma _k^{(t)})$ and $W_j( x;\theta ^{(t)})=\pi _j^{(t)}\phi ( x; \mu _j^{(t)}, \varSigma _j^{(t)})$.

1. *Initialization:* The procedure is initialized nstart times by randomly selecting different initial $\theta ^{(0)}=(\pi _1^{(0)},\ldots ,\pi _k^{(0)}, \mu _1^{(0)},\ldots , \mu _k^{(0)}, \varSigma _1^{(0)},\ldots , \varSigma _k^{(0)})$ sets of parameters. A simple strategy for this initialization is to randomly select $k\times (p+1)$ observations and use them, after splitting them into *k* groups, to compute *k* initial $\mu _j^{(0)}$ centers and *k* initial scatter matrices $\varSigma _j^{(0)}$. It may happen that the initial $\varSigma _j^{(0)}$ matrices do not satisfy the required constraints but the constraints will be imposed in the following iterative step.
2. *Iterative step:*
  $$t \leftarrow t+1$$
  2.1. *Computing observation weights:* From $\theta ^{(t-1)}$, observation weights are computed as
    $$\begin{aligned}&\tau _j(x_i;\theta ^{(t)}) \\&\quad =\left\{ \begin{array}{l} 1 \text { if } W_j( x_i;\theta ^{(t-1)})\\ \qquad =\max \{W_1( x_i;\theta ^{(t-1)}),\ldots ,W_k( x_i;\theta ^{(t-1)})\}\\ 0 \text { if not }\\ \end{array} \right. , \end{aligned}$$
    when maximizing (1) and the associated $H_j$ sets are $H_j^{(t)}=\{i: \tau _j(x_i;\theta ^{(t)}) =1\}.$ On the other hand, when maximizing (2), observation weights are computed as
    $$\begin{aligned} \tau _j(x_i;\theta ^{(t)})= \frac{W_j( x_i;\theta ^{(t-1)})}{\sum _{j=1}^k W_j( x_i;\theta ^{(t-1)})}. \end{aligned}$$
  2.2. *Updating component weights:* From these $\tau _j(x_i;\theta ^{(t)})$ weights, we define
    $$\begin{aligned} n_j= \sum _{i=1}^n \tau _j(x_i;\theta ^{(t)}), \end{aligned}$$
    and the component weights are updated as
    $$\begin{aligned} \pi _j^{(t)} = n_j/n. \end{aligned}$$
  2.3. *Updating location parameters:* Location parameters are updated as
    $$\begin{aligned} \mu _j^{(t)}=\frac{1}{n_j}\sum _{i=1}^n \tau _j(x_i;\theta ^{(t)}) x_i. \end{aligned}$$
  2.4. *Updating scatter matrices:* Updating the scatter matrices $\varSigma _j^{(t)}$ is not so straightforward. As previously commented, the updated scatter matrices are obtained as $\varSigma _j^{(t)}= d_j R_j D_j R_j'$, where these $d_j$, $D_j$ and $R_j$ terms have to be obtained through iterations. Our starting point is the *k* weighted sample covariance matrices defined as
    $$\begin{aligned} S_j=\frac{1}{n_j}\sum _{i=1}^n \tau _j(x_i;\theta ^{(t)})( x_i- \mu _j^{(t)})( x_i- \mu _j^{(t)})'. \end{aligned}$$
    2.4.1 *Initialization:*
      $$u \leftarrow 0$$
      Initially set $d_j=|S_j|^{1/p}$ and $R_j$ as follows:
        rot=V Take $R_j$ as the matrix whose columns are the eigenvectors of the $S_j$ matrices associated with their eigenvalues in decreasing order.
        rot=I Take $$R_1=\cdots =R_k=\textsf {I}_p$$.
        rot=E Take $R_1=\cdots =R_k=R$ where *R* is the matrix whose columns are the eigenvectors of the “pooled” scatter matrix $S=\sum _{j=1}^k \frac{n_j}{n} \frac{1}{d_j}S_j$ associated to its eigenvalues.
    2.4.2 *Iterative part:*
      $$u \leftarrow u+1$$
      2.4.2.1 *Improving the shape* $D_j$ *matrices (needs iterations through* $D_j^{(s)}$):
        (i) $s\leftarrow 0$ and initialize $D_j^{(0)}$ as follows:
          rot=V $$D_j^{(0)}=\texttt {diag}(R_j'S_jR_j)/d_j$$
          rot=I $$D_j^{(0)}=\mathsf {diag}(\sum _{j=1}^k \frac{n_j}{n} \frac{1}{d_j}S_j)$$
          rot=E $$D_j^{(0)}=\mathsf {diag}(\sum _{j=1}^k \frac{n_j}{n} \frac{1}{d_j}R S_j R')$$
        (ii) Take $s\leftarrow s+1$ and apply the “within” constraints as:
          $$\begin{aligned}&\{e_{j1},\ldots ,e_{jp}\} \leftarrow \mathsf {opt.trunc}_{c_{\mathsf {shw}}}\big (\{1\};\\&\quad \{d_{j1}^{(s-1)}, \ldots ,d_{jp}^{(s-1)}\}\big ), \end{aligned}$$
          for $$j=1,\ldots ,k$$.
        (iii) Normalize the elements in $\{e_{j1},\ldots ,e_{jp}\}$ in order to get unit determinant shape matrices by taking $e_{jl} \leftarrow e_{jl}/ \root p \of {\prod _{l=1}^p e_{jl}}$ for $l=1,\ldots ,p$ and $j=1,\ldots ,k$.
        (iv) rot=V case: Consider a permutation $\sigma _j$, which serves to sort the previous elements in decreasing order as $e_{j\sigma _j(1)}\ge \cdots \ge e_{j\sigma _j(p)}$ and take $e_{jl} \leftarrow e_{j\sigma _j(l)}$ for $l=1,\ldots ,p$.
        v) Apply the “between” constraints:
          $$\begin{aligned} \{e_{1l},\ldots ,e_{kl}\} \leftarrow&\mathsf {opt.trunc}_{c_{\mathsf {shb}}} \big (\{n_j\}_{j=1}^k;\\&\{e_{1l},\ldots ,e_{kl}\}\big ) \end{aligned}$$
          for $$l=1,\ldots ,p$$.
        (vi) rot=V case: Undo the order transformations, $e_{jl} \leftarrow e_{j\sigma _j^{-1}(l)}$.
        vii) Update $$D_j^{(s)}=\mathsf {diag}(d_{j1}^{(s)},\ldots ,d_{jp}^{(s)}) \leftarrow \mathsf {diag}(e_{j1},\ldots ,e_{jp})$$
        viii) Go back to ii) if $s < \texttt {iter.max.D}$ and $\varDelta D^{(\cdot )}>\texttt {tol.D}$ or otherwise conclude iterations and finally update
          $$\begin{aligned} D_j \leftarrow D_j^{(s)}. \end{aligned}$$
      2.4.2.2 *Improving the volume* $d_j$ *parameters:*
        Compute $$\nu _1,\ldots ,\nu _k$$ with
        $$\begin{aligned} \nu _j=\mathsf {trace}(D_j^{-1}R_j'S_jR_j)/p \end{aligned}$$
        and update the $d_j$ parameters
        $$\begin{aligned} \{d_1,\ldots ,d_k\} \leftarrow&\mathsf {opt.trunc}_{c_{\mathsf {det}}^{1/p}} \big (\{n_j\}_{j=1}^k;\\&\{\nu _1,\ldots ,\nu _k\}\big ). \end{aligned}$$
      2.4.2.3 *Improving rotations* $R_j$ *matrices:*
        rot=V No change is needed in the $R_j$ matrices
        rot=I Nothing to be done because $R_j=I$
        rot=E *(needs iterations through* $R^{(r)}$): Let $W_j=\frac{n_j}{n} S_j$ and $\omega _j$ is the largest eigenvalue of $W_j$.
          (i) Set $r \leftarrow 0$ and $R^{(0)} \leftarrow R$ for $R_1=\cdots =R_k=R$
          (ii) $$r \leftarrow r+1$$ and
            $$\begin{aligned} F=\sum _{j=1}^k \left( \frac{1}{d_j} D_j^{-1}W_j - \frac{\omega _j}{d_j} D_j^{-1} R^{(r-1)}\right) \end{aligned}$$
            and $F=U\varLambda V$ its singular value decomposition
          (iii) $$R^{(r)} \leftarrow VU$$
          (iv) Go back to ii) if $r < \texttt {iter.max.R}$ and $\varDelta R^{(\cdot )}>\texttt {tol.R}$ or otherwise conclude iterations and finally update
            $$\begin{aligned} R_1=\cdots =R_k \leftarrow R^{(r)}. \end{aligned}$$
      2.4.2.4 Go back to Step 2.4.2 if $u < \texttt {iter.max.} \texttt {dDR}$ and $\max \{\varDelta d, \varDelta D, \varDelta R \} > \texttt {tol.dDR}$.
    2.4.3 Update $$\varSigma _j^{(t)}= d_j R_j D_j R_j'$$
3. Go back to Step 2 if $t< \texttt {iter.max.theta}$ and $\varDelta \theta > \texttt {tol.theta}$.
4. *Evaluate the target function* after applying this iterative process, the associate likelihood, depending on the CEM or EM approach, is computed. The parameters yielding the highest value of this target function are returned as the algorithm’s output.

### Remark 1

As seen in the algorithm, several constants associated with the maximum number of iterations iter.max.theta, iter.max.dDR, iter.max.D and iter. max.R have to be specified. With respect to tolerances, we are using tol.theta, tol.dDR, tol.D and tol.R. When the monitoring parameters $b^{(h)}$ involving several terms, i.e., $b^{(h)}=\{b_1^{(h)},\ldots ,b_k^{(h)}\}$, the relative changes in the *h*-th iteration are measured as

$$\begin{aligned} \varDelta b=\max _{j=1,\ldots ,k} \left\{ ||\mathsf {vec}(b_j^{(h)})-\mathsf {vec}(b_j^{(h-1)})||/|| \mathsf {vec}(b_j^{(h-1)})|| \right\} . \end{aligned}$$

Notice that we use $\mathsf {vec}(\cdot )$ to convert matrices into vectors when needed. The only exception is when monitoring iterated $R^{(h)}$ rotation matrices where we use

$$\begin{aligned} \varDelta R= \frac{\left| p -\mathsf {trace}\left[ ((R^{(h)})'R^{(h-1)})' (R^{(h)} (R^{(h-1)})')\right] \right| }{p}. \end{aligned}$$

### Remark 2

Trivial computational short-cuts can be introduced in limit cases, where any of these constants $c_{\mathsf {det}}$, $c_{\mathsf {shw}}$ and $c_{\mathsf {shb}}$ are chosen equal to 1 or to $\infty$. In those cases, many iterative steps can be avoided. It is also worthwhile to notice that the most computationally demanding version of the algorithm happens when rot=E.

The rationale behind all the steps in this algorithm follows from adaptation of algorithms in the literature. We always try to improve a subset of parameters conditionally on the remaining ones, and, of course fulfilling the required constraints. For instance, the algorithm of Browne and McNicholas (2014) is used in Step 2.4.2.2 and the step in 2.4.2.2 follows from García-Escudero et al. (2020). The“optimal truncation” operator is also used in step 2.4.2.1 to impose the novel constraint in (6).

## Theoretical results

If we assume that $\{x_1,\ldots ,x_n\}$ is a sample from a theoretical probability distribution *P*, a population version of the constrained parsimonious methodology can be defined and existence and consistency results can be proved whenever finite restriction constants $c_{\mathsf {det}}$, $c_{\mathsf {shw}}$ and $c_{\mathsf {shb}}$ are considered.

Given $\theta =(\pi _1,\cdots ,\pi _k,\mu _1,\cdots , \mu _k, \varSigma _1,\cdots ,\varSigma _k)$, we introduce the functions $W_j(x;\theta )=\pi _j \varphi (x;\mu _j,\varSigma _j)$ and $W(x;\theta )=\max \{W_1(x;\theta ),\ldots , W_k(x;\theta )\},$ and the set

$$\begin{aligned} \varTheta _{[c_{\mathsf {det}},c_{\mathsf {shw}},c_{\mathsf {shb}}]}=&\{\theta :\varSigma _1,\ldots ,\varSigma _k\text { satisfy} (4), (5) \text { and }(6) \\&\text { for }c_{\mathsf {det}},c_{\mathsf {shw}} \text { and }c_{\mathsf {shb}}\}. \end{aligned}$$

Theorem 1 provides existence (for both theoretical and sample problem) and consistency result under finite second-order moment conditions.

### Theorem 1

If *P* is not concentrated at *k* points and $E_P \Vert \cdot \Vert ^2 < \infty$

- then there exists some $\theta \in \varTheta _{[c_{\mathsf {det}},c_{\mathsf {shw}},c_{\mathsf {shb}}]}$ such that the maximum of
  10 $$\begin{aligned} E_P\left[ \log \bigg [ \sum _{j=1}^k W_j(\cdot ;\theta ) \bigg ] \right] \end{aligned}$$
  is achieved when $\theta$ is constrained to be in $\varTheta _{[c_{\mathsf {det}},c_{\mathsf {shw}},c_{\mathsf {shb}}]}$.
- then there exists $\theta \in \varTheta _{[c_{\mathsf {det}},c_{\mathsf {shw}},c_{\mathsf {shb}}]}$ such that the maximum of
  11 $$\begin{aligned} E_P\left[ \sum _{j=1}^k z_j(\cdot ;\theta )\log W_j(\cdot ;\theta ) \right] , \end{aligned}$$
  with $z_j(x;\theta )=I\{x: W(x;\theta )=W_j(x;\theta )\},$ is achieved when $\theta$ is constrained to be in $\varTheta _{[c_{\mathsf {det}},c_{\mathsf {shw}},c_{\mathsf {shb}}]}$.

Let us consider an i.i.d. sample $\{x_1,\ldots ,x_n\}$ from the underlying distribution *P* and $P_n=\sum _{i=1}^n \delta _{\{x_i\}}$ the associated empirical distribution. The maximizations (1) and (2) under constraints (4), (5) and (6) when $P=P_n$ reduce exactly to the methodology just presented in Sect. 2. Consequently, Theorem 1 also guarantees the existence of the solution of the empirical problem.

Moreover, a consistency result can be proven for the sequence of empirical maximizers toward the maximizer of the theoretical problem if it is unique (up to a relabelling). Let $\theta _n=(\pi _1^n,\cdots ,\pi _k^n,\mu _1^n,\cdots , \mu _k^n, \varSigma _1^n,\cdots ,\varSigma _k^n) \subset \varTheta _{[c_{\mathsf {det}},c_{\mathsf {shw}},c_{\mathsf {shb}}]}$ denote the sequence of empirical maximizers for the sequence of empirical sample distributions $\{P_n\}_{n=1}^{\infty }$ from *P*. With this notation, Theorem 2 presents the consistency result.

### Theorem 2

Let us assume that *P* is not concentrated at *k* points, that $E_P \Vert \cdot \Vert ^2 < \infty$ and that $\theta _0 \in \varTheta _{[c_{\mathsf {det}},c_{\mathsf {shw}},c_{\mathsf {shb}}]}$ is the unique constrained maximizer of (10), resp. (11), up to a relabelling of the parameters corresponding to each of the *k* components, for *P*. If $\{\theta _n\}_{n=1}^{\infty }$ is a sequence of empirical maximizers of (1), resp. (2), when $\theta _n\in \varTheta _{[c_{\mathsf {det}},c_{\mathsf {shw}},c_{\mathsf {shb}}]}$ then $\theta _n \rightarrow \theta _0$ almost surely.

The proofs of Theorems 1 and 2 derive from similar results in García-Escudero et al. (2020) given that, trivially, we have $\varTheta _{[c_{\mathsf {det}},c_{\mathsf {shw}},c_{\mathsf {shb}}]}\subset \varTheta _{c_1,c_2}$, for $c_1=c_{\mathsf {det}}$ and $c_2=c_{\mathsf {shw}}$, with the same notation for $\varTheta _{c_1,c_2}$ as in García-Escudero et al. (2020). The results in that previous work were, in turn, based on more general theoretical results in García-Escudero et al. (2015) that had been proved for eigenvalues ratio constraints.

### Remark 3

It can be also proven that finite $c_{\mathsf {det}}$ and $c_{\mathsf {shb}}$ values are just enough for existence and consistence results if *P* satisfies $E_P \Vert \cdot \Vert ^2 < \infty$ and *P* is not concentrated at *k* hyperplanes.

## Novel information criteria

In this section, we introduce novel information criteria intended to automatically choose the number of mixture components *k* and $\textsf {pars}=[c_{\textsf {det}}, c_{\textsf {shw}},c_{\textsf {swb}},\textsf {rot}].$ The proposal is to choose

$$\begin{aligned} {[}{\widehat{k}},\widehat{\textsf {pars}}] = \arg \min _{k,\textsf {pars}} \textsf {BIC}[k,\textsf {pars}], \end{aligned}$$

for

12 $$\begin{aligned} \textsf {BIC}[k,\textsf {pars}]=-2 L _k^{\textsf {pars}} +v_k^{\textsf {pars}} \log n, \end{aligned}$$

where $L _k^{\textsf {pars}}$ is the maximum value achieved in the constrained maximization of (2) under constraints defined by $\textsf {pars}$, and where $v_k^{\textsf {pars}}$ is a penalty term defined as:

$$\begin{aligned} v_k^{\textsf {pars}}= & {} \underbrace{kp}_{\text {means}} +\underbrace{k-1}_{\text {weigths}} +\underbrace{(k-1)\left( 1- \frac{1}{c_{\textsf {det}}^{1/p}} \right) +1}_{\text {determinant pars.}} \\&+\underbrace{(p-1)\left( 1 - \frac{1}{c_{\textsf {shw}}}\right) \left[ (k-1)\left( 1 - \frac{1}{c_{\textsf {shb}}}\right) + 1 \right] }_{\text {shape pars.}}\\&+\underbrace{k(\mathsf {rot})\frac{p(p-1)}{2}}_{\text {rotation pars.}}, \end{aligned}$$

with

$$\begin{aligned} k(\mathsf {rot}) = \left\{ \begin{array}{ll} 0 &{} \text {if } \texttt {rot=I} \\ 1 &{} \text {if } \texttt {rot=E} \\ k &{} \text {if } \texttt {rot=V} \end{array} \right. . \end{aligned}$$

Notice that larger values of $c_{\textsf {det}}$, $c_{\textsf {shw}}$ and $c_{\textsf {swb}}$ yield less restricted $\varSigma _j$ scatter matrices, given that more complex models are allowed to be fitted. It is important to note that “model complexity” here does not necessarily correspond to an increased number of parameters, and so “smaller complexity” does not necessarily mean that there are fewer parameters to be interpreted. We also consider a source of complexity for the $\varSigma _j$ matrices which depends on the allowed rotations through $\textsf {rot}$.

The proposal follows a similar philosophy so that introduced in Cerioli et al. (2018). The same arguments in terms of “relative volumes” (as those in Theorem 3.1 in that paper) have been taken into account to derive the previous expression for $v_k^{\textsf {pars}}$. It is easy to see that $v_k^{\textsf {pars}}$ exactly coincides with the number of free parameters for the classical 14 parametrizations which appear as limit cases (restriction constants equal to 1 or tending to $\infty$) reviewed in Table 1. Moreover, the proposal also coincides with the BIC proposal for the “deter-and-shape” constraints previously introduced in García-Escudero et al. (2020) when the constraint (6) is removed by taking $c_{\textsf {shb}}\rightarrow \infty$. In that case, the contribution to $v_k^{\textsf {pars}}$ due to parameters associated to “shape elements” is just

$$\begin{aligned} k(p-1)\left( 1 - \frac{1}{c_{\textsf {shw}}}\right) \end{aligned}$$

and $k(\mathsf {rot})=k$ (no constraints on the rotation matrix).

In this work, we have just focused on the BIC proposal, which is an extension of the MIX-MIX approach in Cerioli et al. (2018). A classification likelihood approach can be also applied by replacing the target function (2) with (1) to define extensions of the MIX-CLA and CLA-CLA approaches (in the spirit of the ICL criterion in Biernacki et al. (2000)).

The minimization of the criterion (12) over all the possible combinations of *k* and $\textsf {pars}$ is not an easy task. In order to circumvent this problem, we just consider powers of 2 for the restriction constants and propose the following procedure:

1. Fix *K* as an upper bound for the maximum number of components and *C* such that $2^{C}$ is large enough that the constraints enforced are not very strict.
2. We first obtain
  13 $$\begin{aligned}&[k^*, c_{\textsf {det}}^*, c_{\textsf {shw}}^*, c_{\textsf {shb}}^*,\textsf {rot}^*] \nonumber \\&=\underset{(k,\textsf {pars}) \in \{1,\ldots ,K\}\times \{1,2^{C-1}\}^3 \times \{\textsf {I},\textsf {E},\textsf {V}\}}{\arg \max } \textsf {BIC}[k,\textsf {pars}]. \end{aligned}$$
  This implies applying the proposed methodology for $K \times 14$ slightly constrained models (and guaranteeing that numerical issues due to singularities are avoided), in correspondence with all the feasible models in Table 1. These $K \times 14$ models need also to be fitted initially as happens with other BIC proposals for parsimonious models. All the other intermediate models (to evaluate) are included within those model fitted with restriction constants equal to $2^{C-1}$.
  This maximization will directly provide our final choice for the number of clusters $k^*$ and for the chosen rotation $\textsf {rot}^*$. Moreover, it also returns our final choices for $c_{\textsf {det}}$, $c_{\textsf {shw}}$ and $c_{\textsf {shb}}$ whenever any of these $c_{\textsf {det}}^*$, $c_{\textsf {shw}}^*$ and $c_{\textsf {shb}}^*$ take the value 1.
3. Constants $c_{\textsf {det}}$, $c_{\textsf {shw}}$ and $c_{\textsf {shb}}$ need to be refined because just upper bounds are initially allowed in Step 1. To perform these refinements, let us obtain
  14 $$\begin{aligned}&[c_{\textsf {det}}^{**},c_{\textsf {shw}}^{**},c_{\textsf {shb}}^{**}] \nonumber \\&\quad =\underset{(c_{\textsf {det}}, c_{\textsf {shw}},c_{\textsf {shb}}) \in {\mathcal {C}}}{\arg \max } \textsf {BIC}[k=k^*, c_{\textsf {det}}, c_{\textsf {shw}},c_{\textsf {shb}},\textsf {rot}\nonumber \\&\quad =\textsf {rot}^*], \end{aligned}$$
  where
  $$\begin{aligned} {\mathcal {C}}=&\big \{2^0,\ldots ,\min \{c_{\textsf {det}}^*,2^{C-1}\}\big \} \\&\times \big \{2^0,\ldots ,\min \{c_{\textsf {shw}}^*,2^{C-1}\}\big \} \\&\times \big \{2^0,\ldots ,\min \{c_{\textsf {shb}}^*,(c_{\textsf {shw}}^*)^{(p-1)/p}, 2^{C-1}\}\big \}. \end{aligned}$$
  We are taking advantage of the initial information about parameters resulting from Step 1 and applying Lemma 1 to reduce notably the number of configurations to be tested.
4. After this process, we finally consider $$[k^*,c_{\textsf {det}}^{**},c_{\textsf {shw}}^{**}, c_{\textsf {shb}}^{**}, \textsf {rot}^*]$$ as a suggestion for $$[{\widehat{k}},\widehat{\textsf {pars}}]$$.

## Simulation study

In this section, we show the advantage of our constrained proposals using simulated datasets. We first consider examples where the number of clusters *k* is assumed to be known (Sect. 6.1). We then show the effectiveness of the novel information criteria for choosing models (Sect. 6.2). As mentioned in the introduction section, we do not claim that the well-established and widely applied proposals considered for comparison in this section are useless, since they clearly have long proven their validity in many scenarios and real data applications. We show examples that highlight the usefulness of the proposed methodology in achieving extra stability.

### Comparison for a fixed number of components

We compare first the performance of the proposed methodology with respect to well-known implementations of the 14 parsimonious model-based clustering methods when assuming a known number of components and the parametrization needed (the VVV parametrization in all cases).

The first example is based on $k=3$ normally distributed components, where the first two coordinates $(X_1,X_2)'$ of these components are generated from bivariate normals with mean parameters equal to $(0,0)'$, $(2,6)'$ and $(6,0)'$ , and the covariances matrices are

$$\begin{aligned} \begin{pmatrix} 2 &{} 0\\ 0 &{} 2 \end{pmatrix} , \begin{pmatrix} 3 &{} 0\\ 0 &{} 1 \end{pmatrix} \text { and } \begin{pmatrix} 1 &{} 0\\ 0 &{} 2 \end{pmatrix}, \end{aligned}$$

respectively. A third independent component $X_3$ is generated from a univariate normal with 0 mean and variance 100, i.e., $X_3\sim N(0,100)$. This simulation scheme is denoted as “lower *p*” case, but we also add an independent fourth coordinate $X_4\sim N(0,100)$ to generate the “higher *p*” case. In the simulation study, we take random samples with $n_1=50$, $n_2=20$ and $n_3=20$ from each component in the “lower *n*” case, and doubled sizes $n_1=100$, $n_2=40$ and $n_3=40$ in the “higher *n*” case.

To explore the effect of the restriction constants, always applying the rot=V case, a three letters notation is used when summarizing the simulation results. The first letter corresponds to the restriction constant chosen in $c_{\textsf {det}}$, the second letter to the constant in $c_{\textsf {shw}}$ and the third letter to the constant $c_{\textsf {shb}}$. In these three letters, we use the letter “C” when the constant defining the constraint is exactly chosen equal to the maximal ratio for this constant computed from the true model generating the data. On the other hand, we use letter “U” when this constant is chosen so high that the procedure is (almost unrestricted) by fixing it to be equal to $10^{10}$ (just to avoid very extreme numerical issues). Letter “D” is used when we double the value for the constant fixed in C, letter “E” when we multiply the constant in C by $2^2$, letter “F” when we multiply by $2^4$ and letter “G” when we multiply by $2^8$.

We always consider the case $k=3$ and rot=V and apply the algorithm in Sect. 3 with nstart$=1000$ and iter.max$=100$. We have included in the simulation study two particular cases, namely, the CCU (that corresponds to the “deter-and-shape” proposal with a large $c_{\textsf {shb}}=10^{10}$), and the CUC (corresponding to a large $c_{\textsf {shw}}=10^{10}$).

The results obtained are compared with those which come from applying the mixture (Browne et al. 2018) and the Mclust (Scrucca et al. 2016) packages in R, when using the VVV parametrization in both cases and when searching for $k=3$ components. We consider two available options for initializing based on the *k*-means method (“mix_km”) and on 1000 random starts (“mix_rs”) when applying the mixture package.

**Fig. 2:**
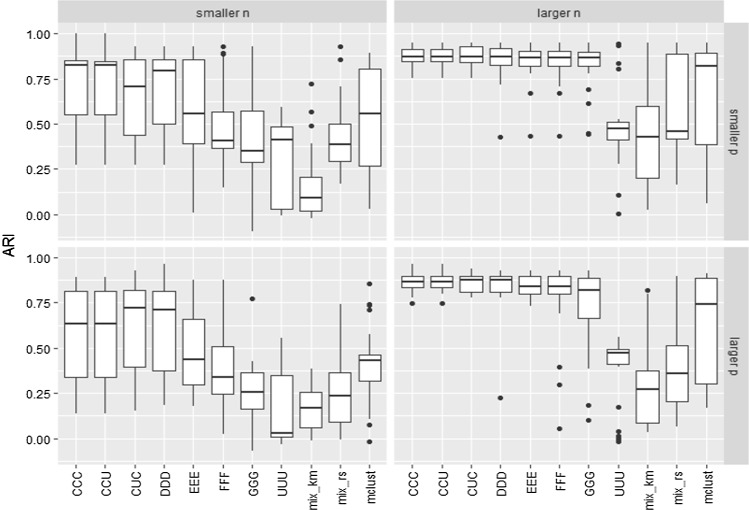
ARI values in the comparative study for the $k=3$ components example

Figure 2 shows the value of the ARI-Adjusted Rand Indexes (Hubert and Arabie 1985) for the obtained partitions, with respect to the true classification, on the same 100 simulated data sets for each of the four possible scenarios depending on the two possible dimensions and two possible sample sizes.

**Fig. 3:**
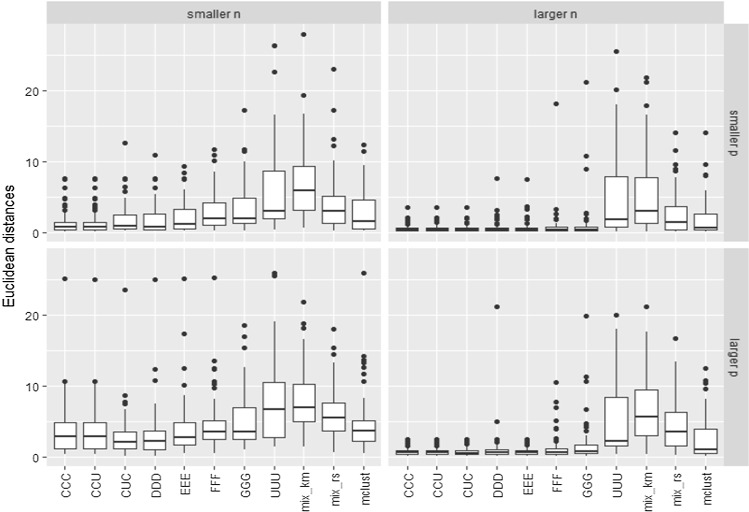
Sum of the Euclidean distances when estimating the true mean vectors in the comparative study for the $k=3$ components example

In order to see the effect on the estimation of the parameters, the Euclidean distances when estimating all the true mean vectors $\mu _1=(0,0,0)'$, $\mu _2=(2,6,0)'$ and $\mu _3=(6,0,0)'$ (lower *p*) and $\mu _1=(0,0,0,0)'$, $\mu _2=(2,6,0,0)'$ and $\mu _3=(6,0,0,0)'$ (higher *p*) are shown in Fig. 3. Relabelling has been applied to match estimators with the estimated location parameters.

**Fig. 4:**
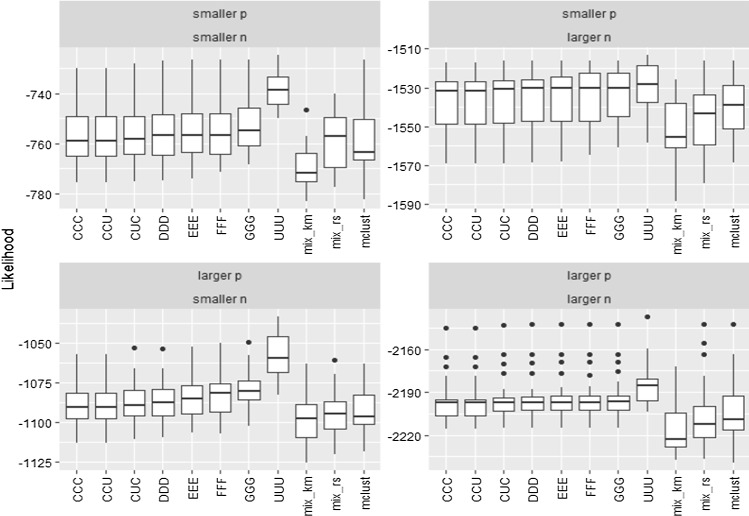
Maximum values achieved when maximizing the target likelihood function for the $k=3$ components example

Figure 4 finally shows the maximum values achieved by the mixture likelihood obtained when maximizing the defining target likelihood function in (2).

As expected, the “higher *n*” cases exhibit clearly better performances. We can also see in Figs. 2 and 3 that the constrained approaches seem to provide higher ARI values and lower estimation errors than their competitors, and that those including letters C and D exhibit the most accurate results among them. We can also see that the least constrained approaches (including letters E, F, G and U) do not provide good results (because stability seems to be lost when increasing the restriction constants), but values of these constants greater than the true ones in C are in general not excessively detrimental. On the other hand, the unconstrained case UUU gives the worst performance. We can also see similar unsatisfactory behavior as in the UUU case when applying the VVV models with the mixture and the Mclust packages. These approaches also considered theoretically a fully unrestricted approach as in the UUU case. However, despite this lack of constraints in the scatter matrices, we observe that the performance of mixture and Mclust depend heavily on the initializing procedure. In this regard, we can see that the initialization based on *k*-means is not satisfactory due to the particular data generation scheme, which is clearly not appropriate for *k*-means.

Even though “mix_rs” is also based on 1000 random initializations, as in our constrained proposals, we can see in Fig. 4 that it does not reach values in the target likelihood so high as those obtained in the UUU case (that could perhaps be even greater if these constants were chosen at values greater than $10^{10}$). Therefore, the type of initializations considered in Step 1 of our algorithm seems to better explore the parametric space than the initializations in “mix_rs”. The same happens with the initializations provided by *k*-means or by the initializing procedure based on hierarchical model-based clustering applied by Mclust. The initializations can be useful to avoid spurious solutions, but it is also important to note that they are not striving to maximize the target likelihood function and they clearly influence the performance of the methodology. Figure 4 also shows how the target likelihoods steadily increase when increasing the values of the restriction constants (C, D, E, F, G and U) and this could serve to understand the degree of stability provided by constraints and help us to achieve smooth transitions between models.

**Fig. 5:**
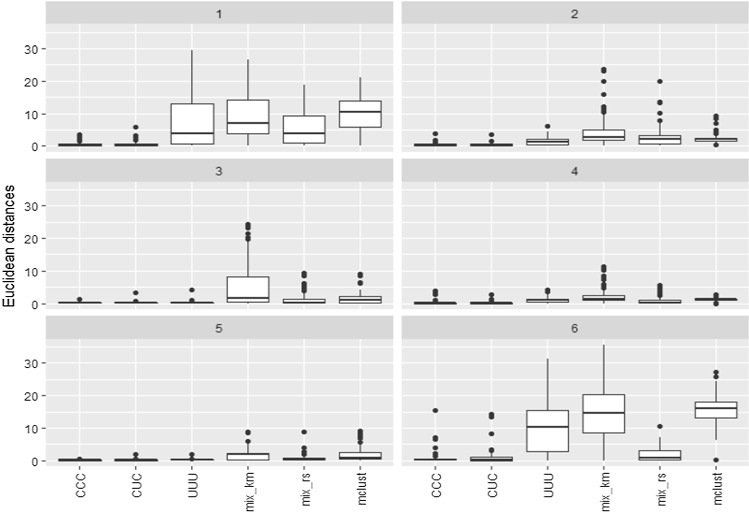
Euclidean distances when estimating the true mean vectors in the comparative study for the $k=6$ components example. Each panel corresponds to one of the components

We briefly present another example with a higher $k=6$ number of components. The example starts from a bivariate mixture of six spherical normally distributed components with the same scatter and component sizes equal to $n_1=23$, $n_2=36$, $n_3=93$, $n_4=38$, $n_5=123$ and $n_6=12$ with the following mean vectors: $(-4.5,3.6)'$, $(0.40,3.6)'$, $(-4.4,-1)'$, $(9.2,-1)'$, $(0.4,-1)'$, and $(9.2,3.6)'$. We add a third dimension by using an independent variable $X_3\sim N(0,100)$, which makes these clusters become elongated.. A simulation study, completely analogous to the one previously described is considered. Figure 5 provides the Euclidean distances when estimating the corresponding six means of the components individually.

We can see that the constrained approach seems to provide better results also in this $k=6$ example.

### Comparison when choosing number of components and models

We also compare the performance of the novel information criteria introduced in Sect. 5 with respect to the BIC procedures resulting from the application of the mixture package (Browne et al. 2018). With this aim in mind, we simulate 100 samples of size $n=200$ in dimension $p=10$ for each of the 14 classical parsimonious models. To be more precise, each sample is generated from a $k=3$ components mixture where $\mu _1$, $\mu _2$ and $\mu _3$ and $\varSigma _1$, $\varSigma _2$ and $\varSigma _3$ are randomly generated parameters in such a way that the covariance matrices satisfy the specific model constraints and also a prefixed overlap rate equal to 0.05. That overlap is achieved by applying the extension of the MixSim method of Maitra and Melnykov (2010) given in Riani et al. (2015). Given two clusters *j* and *l* obtained from normal densities $\phi (\cdot ;\mu _j, \varSigma _j )$ and $\phi (\cdot ;\mu _l, \varSigma _l)$, with probabilities of occurrence $\pi _j$ and $\pi _l$, the overlap between groups *j* and *l* is defined as the sum of the two misclassification probabilities $w_{jl} = w_{j|l} + w_{l| j}$ where $w_{j|l} = P[\pi _l\phi (X;\mu _l, \varSigma _l) < \pi _j\phi (X;\mu _j, \varSigma _j)]$. The average overlap is the sum of the off-diagonal elements of the matrix of the misclassification probabilities $w_{j|l}$ divided by $k(k - 1)/2$. Note that when we say that the covariance matrices satisfy the model constraints, we mean that we ensure that the $\varSigma _1$, $\varSigma _2$ and $\varSigma _3$ matrices do exactly satisfy the constrained models with the values of $c_{\textsf {det}}$, $c_{\textsf {shw}}$ and $c_{\textsf {swb}}$ as in Table 1 but replacing the values of “$\infty$” in that table by values equal to 100 in the case of $c_{\textsf {det}}$ or $c_{\textsf {shw}}$ and by a value equal to 10 in the case of $c_{\textsf {swb}}$. The mixture components weights are always $\pi _1=\pi _2=\pi _3=1/3$.

**Fig. 6:**
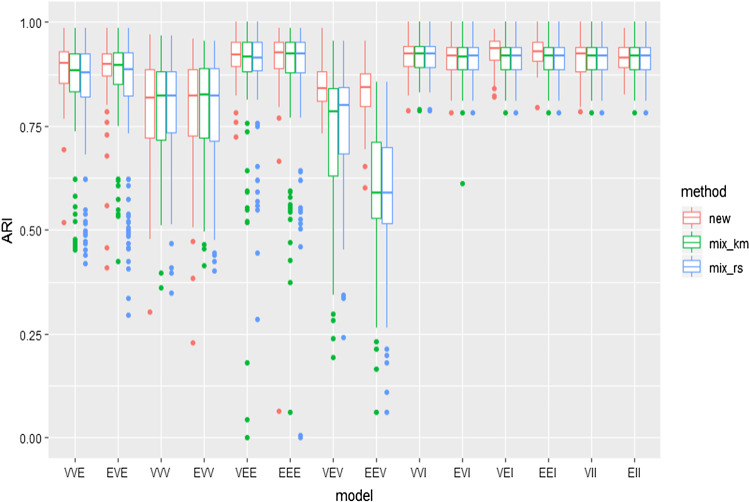
ARI values for the simulated data sets when applying the novel information criteria and the BIC procedure in the mixture package

Figure 6 shows the ARI indexes between the true partition and the partition obtained from the fitted mixture suggested by the BIC-type information criterion. In this figure, we use the notation “new” for the results associated with the new proposed methodology and “mix_km” for those with the mixture package when initialized with *k*-means and “mix_rs” when initialized by using random starts (the same number of random starts nstart as in “new” are considered).

**Fig. 7:**
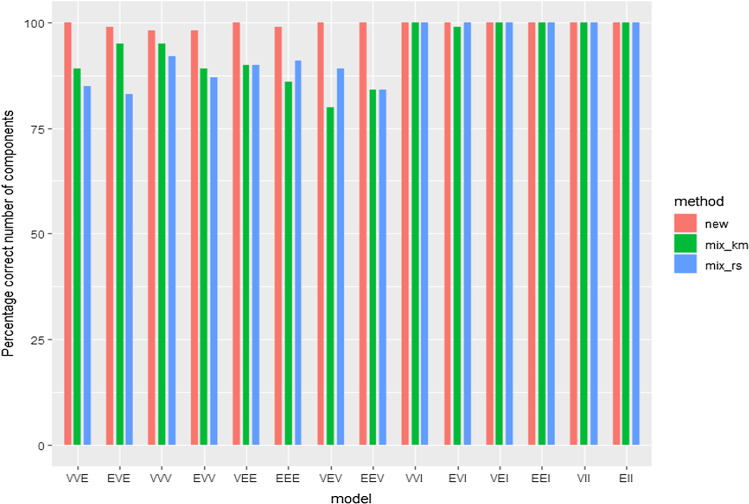
Proportion of times that the true number of components $k=3$ is determined by the information criteria

Figure 7 shows the proportion of times that the true number of components, $k=3$, is determined by the BIC-type information criteria.

We can see in these two figures that the BIC methodology in the mixture package is reasonably able to recover the right number of components and the true data generating mechanism. However, the comparison is clearer in Fig. 7 when looking at the proportion of times that the true number of components is detected. We see that better results are obtained when applying the new constrained approaches. Of course, those differences are not so noticeable for the most constrained (VVI, EVI, ..., EII) models, where no great advantages can be achieved by restricting even more.

The improvement is more clearly seen in Fig. 7 than in Fig. 6, perhaps because spurious components, made up of few observations, do not significantly modify the ARI, even though they change the number of components detected. This wrong determination of the number of components may of course be problematic, when interpreting results. Note also that constrained approaches seem to avoid partitions exhibiting very low ARI values (outliers outside whiskers in Fig. 6).

## Real data example: COVID data

**Fig. 8:**
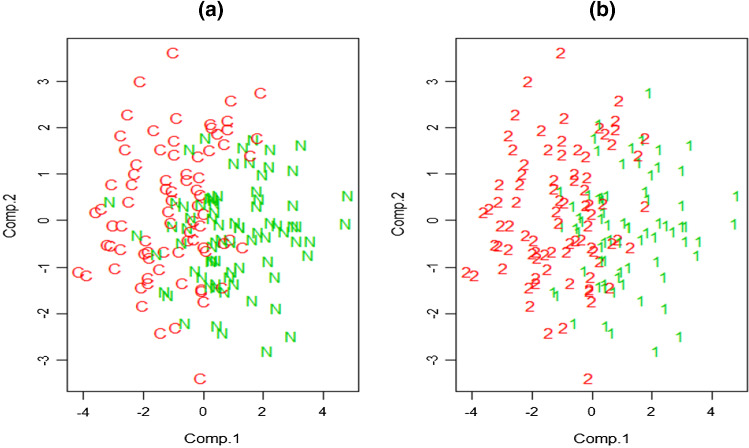
**a** Doctor-based “true” classification for the COVID data set with COVID patients denoted with C symbols and non-COVID by N symbols with observations represented in the first two principal components. **b** Clustering results of the constrained parsimonious model-based clustering proposal with parameters chosen from the new BIC procedure

The example is inspired by the analysis of a real data set on the SARS-CoV-2 symptoms kindly provided to us by the ASL3 Genovese Hospital. Measurements on six variables $x_1=$ “heart rate (the number of beats the heart per minute)”, $x_2=$ “Oxygen Uptake Efficiency Slope (index of functional reserve derived from the logarithmic relation between oxygen uptake and minute ventilation during incremental exercise),” $x_3=$ “watts (reached by the patient during the stress test on a cycle ergometer (stationary bike) at the aerobic threshold, that is, when the patient ’begins to struggle’),” $x_4=$ “watts peak (watts reached at maximum effort (during exercise test on exercise bike),” $x_5=$ “value of the maximum repetition (maximum force of muscle contraction of the quadriceps femoris of the dominant limb expressed in kg)” and $x_6=$ “previous variable corrected on the subject (in relation to the patient’s weight)” on 79 COVID patients and 77 non-COVID ones. Figure 8a shows the (supposedly) true classification, as provided by the doctors. Data have been collected by “Post-COVID Outpatient Rehabilitation Center ASL3 Liguria Region Health System” and approved by the Ethics Committee of Liguria region (Italy).

We will apply the (unsupervised) constrained model-based clustering approach to see if something close to the doctor’s classification partition is achieved. We will use the modified BIC approach described in Sect. 5 to determine the underlying number of clusters and the set of constraints to be imposed.

The proposed BIC approach described in Sect. 5 is applied with $K=5$ and $C=8$ (i.e., $2^{C-1}=128$). In the maximization of (13), after fitting $5\times 14$ models, we obtain $k^*=2$, $c_{\textsf {det}}^*=1$, $c_{\textsf {shw}}^*=128$, $c_{\textsf {shb}}^*=1$ and $\textsf {rot}^*\textsf {=E}$. Afterwards, we perform the maximization in (14) where ${\mathcal {C}}=1\times \{2^0,2^1,\ldots ,2^{7-1}\}\times 1$. This means that we need to obtain

$$\begin{aligned} c_{\textsf {shw}}^{**}&= \underset{ c_{\textsf {shw}} \in \{1,2,\ldots ,128\} }{\arg \max } \textsf {BIC}[k=2, c_{\textsf {det}}=1, c_{\textsf {shw}},c_{\textsf {shb}}\\&=1,\textsf {rot}=\textsf {E}], \end{aligned}$$

(as we directly have $$c_{\textsf {det}}^{**}=1$$ and $$c_{\textsf {shb}}^{**}=1$$). Our BIC proposal suggests $$[{\widehat{k}},\widehat{\textsf {pars}}]=[2,1,128,1,\textsf {E}]$$. This is a quite constrained solution where only the “within” components shape elements are left notably unrestricted (the value of $$c_{\textsf {shw}}$$ is such that only a slightly constrained ratio is considered). The associated partition is shown in Fig. 8b and exhibits an ARI index with respect to the “true” doctor’s classification equal to 0.5891.

On the other hand, the BIC approach implemented through function gpcm() in the mixture package in R (Browne et al. 2018) suggests $k=2$ but the EEE parameterization results in an ARI equal to 0.0117 with respect to the doctor’s suggested partition. This result is obtained when considering a *k*-means type initialization but the results do not seem to improve when considering random initializations. The results, for this particular data set, do not improve when we apply the BIC criterion provided by the Mclust package (Scrucca et al. 2016) that only suggests one $k=1$ component for this data set. The VEE parameterization is suggested when considering Mclust’s BIC criterion but restricted to models with $k=2$, which yields a ARI=0.0414 that is notably smaller that the 0.5891 achieved when applying the proposed methodology with the new BIC proposal.

## Conclusions and further directions

We have introduced a new methodology for constrained parsimonious model-based clustering that depends on three restriction constants $c_{\textsf {det}}$, $c_{\textsf {shw}}$ and $c_{\textsf {shb}}$ and on fixing a particular type rot of rotations. The methodology provides a smooth transition among the well-known 14 parsimonious models that are commonly applied in model-based clustering when assuming normality for the components. The proposed constraints result in mathematically well-defined problems and provide extra control on the covariance matrices of the fitted components. Novel information criteria have been introduced to help the user in providing sensible choices for all the tuning decisions.

There are many open research lines related to this new approach. For instance, dealing with computational aspects could still be needed to speed up the procedures. Although MATLAB code for its practical application is now available, we are developing a more dedicated and easy to apply implementation within the FSDA MATLAB toolbox (Riani et al. 2012). This implementation will hopefully include more elaborate graphical and numerical tools in helping to determine and explore the solutions obtained when moving all the involved parameters in the spirit of Cerioli et al. (2018). With that aim, stability and ARI distances among partitions could be taken into account in order to derive a reduced (and ranked) list of sensible partitions and also graphical summaries as the “car-bike plots.” The methodology can be also adapted to include “trimming” to introduce new robust model-based clustering approaches.

## Appendix A: Proof of Lemma 1

Let us consider $\xi _{j,l}=\log \gamma _{jl}$ such that

$$\begin{aligned} \xi _{j,1}\ge \cdots \ge \xi _{j,l}\ge \cdots \ge \xi _{j,p}, \end{aligned}$$

as in (7), under the constraint

15 $$\begin{aligned} \sum _{l=1}^p \xi _{j,l}=0, \end{aligned}$$

(given that $\prod _{l=1}^p \gamma _{jl}=1$). Constraints (5), after log-transformation, reduce to $\xi _{j,l}-\xi _{j,l'}\le \log c_{\mathsf {shw}}$ for any $l,l' \in \{1,\ldots ,p\}$ for a fixed *j*.

It is not difficult to see that the maximal possible difference is achieved with repeated $\xi _{j,l}$ values and setting that maximal difference equal to $\log c_{\mathsf {shw}}$. In that case, the most extreme difference is for *j* and $j'$ when

$$\begin{aligned} \xi _{j,1}=\cdots =\xi _{j,l}=\xi _{j,p}+\log c_{\mathsf {shw}}\text { and }\xi _{j,l+1}=\cdots =\xi _{j,p} \end{aligned}$$

and

$$\begin{aligned}&\xi _{j',1}=\cdots =\xi _{j',l-1}=\xi _{j',p}+\log c_{\mathsf {shw}}\text { and }\\&\xi _{j',l}=\cdots =\xi _{j',p}. \end{aligned}$$

By taking into account the zero sum condition (15), we have $0=p\xi _{j,p}+l \log c_{\mathsf {shw}}$ and $0=p\xi _{j',p}+(l-1) \log c_{\mathsf {shw}}$, for these two configurations. Therefore,

$$\begin{aligned} p(\xi _{j,l}-\xi _{j',l})= & {} (p\xi _{j,l}-(p\xi _{j,p}+l \log c_{\mathsf {shw}}))\\&-(p\xi _{j',l}-(p\xi _{j',p}+(l-1) \log c_{\mathsf {shw}}))\\= & {} (p-1) \log c_{\mathsf {shw}}, \end{aligned}$$

where we have used that $\xi _{j,l}-\xi _{j,p}=\log c_{\mathsf {shw}}$ and that $\xi _{j',l}-\xi _{j',p}=0$. Consequently, $\xi _{j,l}-\xi _{j',l}= \frac{p-1}{p} \log c_{\mathsf {shw}}$ for that most extreme possible difference and so (8) is just proven after exponentiation.

## Appendix B: “Optimal truncation” operator

For sake of completeness, we review the “optimal truncation” procedure (Fritz et al. 2013) that has been extensively used in the algorithm in Sect. 3.

Given a $d\ge 0$ and a fixed restriction constant $c\ge 1$, we introduce the *m*-truncated value is defined as

$$\begin{aligned} d^m = \left\{ \begin{array}{ll} d&{}\text { if } d\in [ m,cm] \\ m&{}\text { if }d<m \\ cm&{}\text { if } d>cm \end{array} \right. . \end{aligned}$$

Given $\{n_j\}_{j=1}^J \in {\mathbb {N}}^J$ and $\{ d_{j1},\ldots ,d_{jL}\}_{j=1}^J \in [0,\infty )^{J\times L}$, we define that operator as

$$\begin{aligned} \mathsf {opt.trunc}_c\big (\{n_j\}_{j=1}^J;\{ d_{j1},\ldots ,d_{jL}\}_{j=1}^J\big ), \end{aligned}$$

which returns $\{d_{j1}^{*},\ldots ,d_{jL}^{*}\}_{j=1}^J \in [0,\infty )^{J\times L}$ with $d_{jl}^{*}=d_{jl}^{m_{\text {opt}}}$ for $m_{\text {opt}}$ being the optimal threshold value obtained as

16 $$\begin{aligned} m_{\text {opt}}=\arg \min _m \sum \limits _{j=1}^{J}n_j \sum \limits _{l=1}^{L}\left( \log \left( d_{jl}^m\right) + \frac{ d_{jl}}{d_{jl}^m} \right) . \end{aligned}$$

Obtaining that optimal threshold value only requires the maximization of a real-valued function and $m_{\text {opt}}$ can be efficiently obtained by performing only $2\cdot J\cdot L +1$ evaluations (Fritz et al. 2013) of (16) through a procedure which can be fully vectorized.

## References

[CR1] Banfield JD, Raftery AE (1993). Model-based Gaussian and non-Gaussian clustering. Biometrics.

[CR2] Biernacki C, Celeux G, Govaert G (2000). Assessing a mixture model for clustering with the integrated completed likelihood. IEEE Trans. Pattern. Anal. Mach. Intell..

[CR3] Biernacki, C., Celeux, G., Govaert, G.: Choosing starting values for the em algorithm for getting the highest likelihood in multivariate gaussian mixture models. Comput. Stat. Data Anal. **41**, 561–575 (2003)

[CR4] Browne R, McNicholas P (2014). Estimating common principal components in high dimensions. Adv. Data. Anal. Classif..

[CR5] Browne RP, ElSherbiny A, McNicholas PD (2018). mixture: mixture models for clustering and classification. R Package Version.

[CR6] Celeux G, Govaert G (1995). Gaussian parsimonious clustering models. Pattern Recognit..

[CR7] Cerioli A, García-Escudero LA, Mayo-Iscar A, Riani M (2018). Finding the number of normal groups in model-based clustering via constrained likelihoods. J. Comput. Graph. Stat..

[CR8] Day N (1969). Estimating the components of a mixture of normal distributions. Biometrika.

[CR9] Fritz H, García-Escudero LA, Mayo-Iscar A (2013). A fast algorithm for robust constrained clustering. Comput. Stat. Data Anal..

[CR10] Gallegos MT, Ritter G (2018). Probabilistic clustering via pareto solutions and significance tests. Adv. Data Anal. Classif..

[CR11] García-Escudero LA, Gordaliza A, Matrán C, Mayo-Iscar A (2015). Avoiding spurious local maximizers in mixture modeling. Stat. Comput..

[CR12] García-Escudero LA, Gordaliza A, Greselin F, Ingrassia S, Mayo-Iscar A (2018). Eigenvalues and constraints in mixture modeling: geometric and computational issues. Adv. Data Anal. Classif..

[CR13] García-Escudero LA, Mayo-Iscar A, Riani M (2020). Model-based clustering with determinant-and-shape constraint. Stat. Comput..

[CR14] Hathaway R (1985). A constrained formulation of maximum likelihood estimation for normal mixture distributions. Ann. Stat..

[CR15] Hubert L, Arabie P (1985). Comparing partitions. J. Classif..

[CR16] Kiefer J, Wolfowitz J (1956). Consistency of the maximum likelihood estimator in the presence of infinitely many incidental parameters. Ann. Math. Stat..

[CR17] Maitra R, Melnykov V (2010). Simulating data to study performance of finite mixture modeling and clustering algorithms. J. Comput. Graph. Stat..

[CR18] McLachlan G, Peel D (2000). Finite Mixture Models.

[CR19] Meng X, Rubin D (1993). Maximum likelihood estimation via the ECM algorithm: a general framework. Biometrika.

[CR20] Riani M, Perrotta D, Torti F (2012). FSDA: a Matlab toolbox for robust analysis and interactive data exploration. Chemometr. Intell. Lab. Syst..

[CR21] Riani M, Cerioli A, Perrotta D, Torti F (2015). Simulating mixtures of multivariate data with fixed cluster overlap in FSDA library. Adv. Data Anal. Classif..

[CR22] Scrucca L, Fop M, Murphy TB, Raftery AE (2016). mclust 5: clustering, classification and density estimation using Gaussian finite mixture models. R J..

